# Cinacalcet in Pediatric and Adolescent Chronic Kidney Disease: A Single-Center Experience

**DOI:** 10.1097/MD.0000000000000401

**Published:** 2015-01-16

**Authors:** Abdulla A. Alharthi, Naglaa M. Kamal, Mohamed W. Abukhatwah, Laila M. Sherief

**Affiliations:** From the Faculty of Medicine, Taif University, Al Hada Armed Forces Hospital, Taif, KSA (AAA); Faculty of Medicine, Cairo University, Cairo, Egypt, Al Hada Armed Forces Hospital, Taif, KSA (NMK); Al Hada Armed Forces Hospital, Taif, KSA (MWA); and Faculty of Medicine, Zagazig University, Egypt (LMS).

## Abstract

Cinacalcet, a calcimimetic drug, has been shown to be efficacious in adult chronic kidney disease (CKD) patients; however, it was not fully studied in pediatric CKD patients. We aimed at assessing the effect of cinacalcet on intact parathyroid hormone (iPTH) secretion in children with CKD-4/5 with iPTH consistently ≥ 300 pg/mL refractory to conventional treatment.

This is a prospective cohort analysis of 28 children with uncontrolled hyper-parathyroidism secondary to stage 4 and 5 CKD admitted to a tertiary center during the period from April 2012 to April 2014.

Twenty-eight patients with CKD-4/5 were assessed prospectively regarding bone biochemistry, renal ultrasonography, serum iPTH level, and medications. Patients were classified into 3 groups: group 1, 6 patients with CKD-4 on supplemental and supportive therapy; group 2, 6 patients with CKD-5 on hemodialysis and; group 3, 16 patients with CKD-5 on automated peritoneal dialysis. Patients were between the ages of 9 months and 18 years on commencing cinacalcet at doses of 0.5 to 1.5 mg/kg.

All patients showed at least a 60% reduction in iPTH (60%–97%). Highly significant reduction in iPTH and serum alkaline phosphatase levels was detected post-cinacalcet. The serum calcium (Ca), phosphate (P), and Ca × P product were unaffected. Treatment was well tolerated with no hypophosphatemia, hypocalcemia, or other adverse effects almost in all patients.

Cinacalcet use was proven safe for all pediatric and adolescent patients with CKD-4/5 during the study period, and at the same time most of the patients reached the suggested iPTH target values

## INTRODUCTION

Chronic kidney disease (CKD) is accompanied by profound disturbances in calcium (Ca), phosphate (P), vitamin D (Vit D), and intact parathyroid hormone (iPTH) homeostasis that play a crucial role in the pathophysiology of renal bone disease.^1^ Renal osteodystrophy encompasses 3 distinct histological entities: high turnover, which includes osteitis fibrosa cystica and is associated with high iPTH levels; adynamic bone disease associated with low iPTH levels; and mixed.^2^

The control of P and Ca metabolism is one of the objectives in an adequate continuous renal replacement therapy. Treatment of renal osteodystrophy is essential for prevention of musculoskeletal disorders, cardiovascular calcifications, and ultimately for reduction of morbidity and mortality rates.^3–5^

The United Kingdom (UK) Renal Association Guidelines and National Kidney Foundation Disease Outcomes Quality Initiative (K/DOQI) guidelines were produced with the aim of reducing the consequences of secondary hyper-parathyroidism by establishing targets for biochemical indices including iPTH, Ca, P levels, and Ca × P product.^2^ However, these 2 sets of guidelines are very different with regard to recommendations for iPTH.^2^

The K/DOQI guideline recommendations are the following:

1. Serum P should be maintained at or above the age-appropriate lower limit (EVIDENCE), but not higher than the age-appropriate upper limits (OPINION) for CKD-4 and between 4 and 6 mg/dL (1.29–1.93 mmol/L) in children 1 to 12 years of age and between 3.5 and 5.5 mg/dL (1.13–1.78 mmol/L) in adolescents with CKD-5 (EVIDENCE).^6^
2. Serum levels of corrected total Ca should be maintained within the normal range for the laboratory use in CKD-4 (EVIDENCE) and within the normal range for the laboratory use (8.8–9.7 mg/dL [2.20–2.37 mmol/L]), and preferably toward the lower end (OPINION) for CKD-5.^6^
3. The serum Ca × P should be maintained at <55 mg^2^/dL^2^ in adolescents >12 years and <65 mg^2^/dL^2^ in younger children (OPINION).^6^
4. Serum iPTH should be between 70 and 110 for CKD-4 and between 200 and 300 pg/mL for CKD-5.^6^
5. Serum Ca and P should be measured at least every 3 months in CKD-4 and at least monthly in CKD-5, whereas serum iPTH should be measured at least every 3 months for CKD-4/5 pediatric patients.^6^

It is found that secondary hyperparathyroidism frequently develops during stages 3 and 4 of CKD.^7^ Treatment options aimed at suppressing iPTH currently include dietary P restrictions, P binding, and supplementation with active Vit D and Ca. Dialysis provides additional means of P removal as well as helping to control acid–base balance and removes uremic toxins detrimental to bone health.^8^ Dietary P restriction is problematic in children because of the consequent reduction in protein intake needed to achieve the intended results.^9^ Existing treatment strategies (active Vit D, Ca supplements, Ca-containing P binders) have the potential to increase serum levels of Ca. Ca and P at high levels are both independent predictors of adverse cardiovascular outcomes as a result of metastatic calcification.^10–12^ Therefore, there has been a more recent focus on developing treatments that will suppress iPTH secretion and minimize elevation of these minerals. The Ca-sensing receptor (CaSR), first identified and cloned in 1994,^13^ is thought to be the prime regulator of iPTHsecretion^14–15^ and as such has become the target for newer therapies in the treatment of both primary and secondary hyperparathyroidism.^16,17^ Cinacalcet is a type II calcimimetic that acts as an allosteric modulator of the CaSR on the parathyroid cells to increase its sensitivity to extracellular Ca ions and diminish iPTH secretion in response.^18^ Cinacalcet shifts the set point for Ca-regulated iPTH secretion to the left leading to a reduction in iPTH secretion.^7^

Cinacalcet has been shown to be efficacious in adult CKD patients, allowing improved control of serum biochemistry as specified by K/DOQI,^19^ as well as reducing rates of parathyroidectomy, pathological fracture, and hospital admissions related to cardiovascular complaints.^20^ Current UK National Institute for Health and Clinical Excellence guidance^21^ recommends the use of cinacalcet to treat refractory secondary hyperparathyroidism in patients with end-stage renal disease (including those with calciphylaxis) only in those who have very uncontrolled plasma levels of iPTH consistently >300 pg/mL and refractory to standard therapy, and normal or high adjusted serum Ca level, and in whom surgical parathyroidectomy is contraindicated in that the risks of surgery are considered to outweigh the benefits.” Contraindications for use include a low baseline serum-corrected Ca (<2.1 mmol/L).^2^

We present our experience of the continuous use of cinacalcet for up to 24 months in 28 CKD pediatric patients with uncontrolled hyperparathyroidism (iPTH >300 pg/mL) despite the optimization of conventional management.

To our knowledge, the present study is the first to date to be done on cinacalcet on Saudi andArab children. It is the largest study to date carried on pediatric population suffering from refractory hyperparathyroidism secondary to CKD-5 and the first one carried on CKD-4 with uncontrolled hyperparathyroidism in pediatrics. It is also unique in including the youngest patient receiving cinacalcet (age 9 months at time of inclusion) and in being the longest in duration (24 months) after that carried out by Platt et al^2^ who followed one of his 6 pediatric CKD patients for a longer duration, that is, 36 months.

## PATIENTS AND METHODS

We carried out a prospective, open-label, single-arm interventional study over a period of 24 months; April 2012 to April 2014; on 28 pediatric patients suffering from CKD-4/5 selected from those attending Al-Hada Armed Forces Hospital’ Pediatric department (nephrology clinic, peritoneal dialysis clinic, hemodialysis [HD] unit, and inpatient ward), Taif, Kingdom of Saudi Arabia (KSA), according to the following inclusion and exclusion criteria:

Key inclusion criteria:

1. Age: children and adolescents up to the age of 18 years.
2. Mean baseline iPTH ≥300 pg/mL despite maximum conventional treatment for at least 3 months.
3. Mean baseline corrected serum Ca ≥8.4 mg/dL.
4. Ca × P product ≥65 mg^2^/dL^2^.
5. Those on HD or automated peritoneal dialysis (APD) for >6 months.

Key exclusion criteria:

1. Serum Ca <8.4 mg/dL.
2. Patient with seizure disorder maintained on anti-convulsant treatment as hypocalcemia, which might be caused by cinacalcet, lower the threshold for seizures in these patients.
3. Patients with hepatic impairment as cinacalcet is metabolized in the liver.
4. Patients with hypersensitivity to cinacalcet.

The study was approved by the research and ethical committees of Al-Hada Armed Forces Hospital. The caregivers of the included patients signed a written informed consent for contribution of their children in the present study.

The study aimed at seeking evidence that those patients would benefit from treatment with cinacalcet by achieving the target iPTH level as recommended by K/DOQI clinical guidelines.

They were classified into 3 groups: group 1, 6 patients with CKD-4; group 2, 6 patients with CKD-5 on HD; and group 3, 16 patients with CKD-5 on APD. The HD patients were receiving 3 sessions a week, 4 h per session, whereas those on APD were receiving dialysis for 12 h/d.

All patients were subjected to full history-taking and thorough clinical examination with special concern on growth parameters plotted on growth percentiles.

The results of serum (s) blood urea nitrogen (BUN), s.Creatinine, s.Ca, s.P, s.iPTH, Ca × P product, Vit D, s.alkaline phosphatase (s.Alk.P), glomerular filtration rate (GFR) estimation calculated using Schwartz's formula, 24-hour urinary (u) proteins, u.albumin, u.Ca, u.P, u.creatinine for those with residual renal function (CKD-4), renal ultrasound (U/S), and echocardiography 3 months before cinacalcet treatment were recorded. The laboratory tests were then done on monthly basis after starting cinacalcet treatment, whereas renal U/S and echocardiography were done every 3 months.

There is no standard dose for Cinacalcet in Pediatrics. We started it at a dose of 0.5 mg/kg/d and titrated the dose sequentially every 2 weeks up to a maximum of 1.5 mg/kg/d until the end point is achieved. Cinacalcet was started along with other conventional therapy for CKD (alfacalcidol, P binders, and Ca supplements) in all patients.

The end-point was defined as achievement of the recommendations of the K/DOQI guidelines, namely iPTH <150 to 300 pg/mL in dialysis patients and 70 to 110 pg/mL in CKD-4 patients, Ca 8.4 to 9.5 mg/dL, P 3.5 to 5.5 mg/dL, and Ca × P product <55 mg^2^/dL^2^.

### Statistics

Data management and analysis were performed using Statistical Analysis Systems, SAS version 8.02. Numerical data were summarized using means and standard deviations. Categorical data were summarized as counts and percentages. Differences between 2 groups with respect to numeric variables were tested using the Student *t* test. Chi-square test was used to compare groups with respect to categorical data. *P* value <0.05 was considered significant.

## RESULTS

Before treatment with cinacalcet, all patients had biochemical evidence of high-turnover bone disease, with s.iPTH values consistently >300 pg/mL despite adjustment of all standard medications with failure of control of high s.iPTH, and in the majority of cases, hypercalcemia predating the introduction of cinacalcet had prevented dose escalation of Ca-containing P binders and Vit D analogues.

All patients were maintained on cinacalcet plus combinations of conventional CKD medications (alfacalcidol, Ca carbonate, Sevelamer HCL [Renagel], and cholecalciferol).

The duration of cinacalcet management varied between 3 and 24 months depending on the time needed to achieve the end-point of treatment. The starting dose of cinacalcet for all patients was 0.5 mg/kg/d, which was titrated sequentially every 1 month according to laboratory results until end-point is achieved or adverse event is observed or a maximum dose of 1.5 mg/kg/d is reached. The end-point was achieved early within 6 months on low dose of 0.5 mg/kg/d in cases of CKD-4, whereas those with CKD-5 required higher duration of management up to 12 to 24 months on higher doses of 1 to 1.5 mg/kg/d. Nine patients with CKD-5 reached the maximum dose (1.5 mg/kg/d) and duration (24 months) designed for the study and still did not achieve the end-point. We planned to continue the same plan of management in those 9 patients with plan to release their final results in future follow-up research.

Some of those who achieved the end-point were maintained on low-dose cinacalcet ≤0.5 mg/kg/d on daily, alternate day, or twice weekly therapy to prevent rebound hyperparathyroidism after discontinuation of cinacalcet.

Cinacalcet use had little impact on reducing the total number or doses of conventional CKD medications, especially active Vit D, which was continued throughout the study to guard against hypocalcemia.

The demographic data of the included patients at the start of treatment as well as s.Ca, s.P, and s.iPTH values pre-cinacalcet treatment are shown in Table 1.

**TABLE 1:**
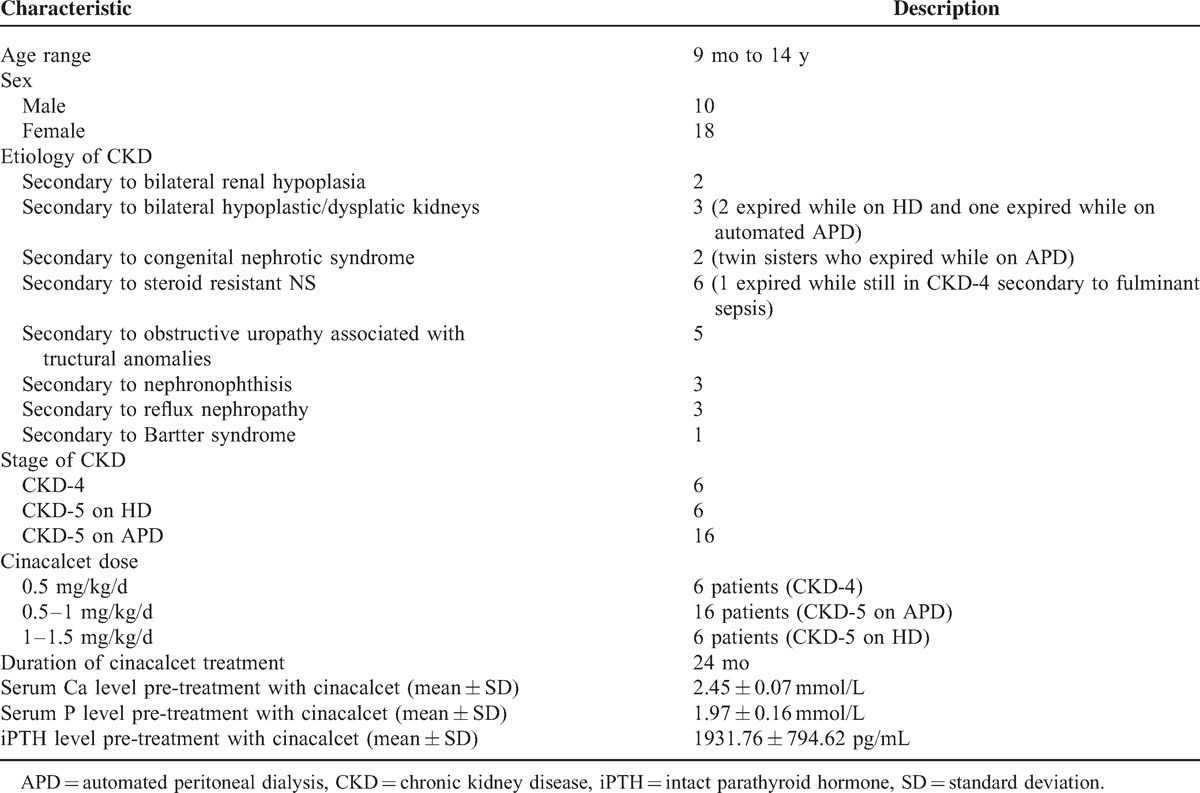
Demographic and Clinical Characteristics of Our Patients

**Figure 1:**
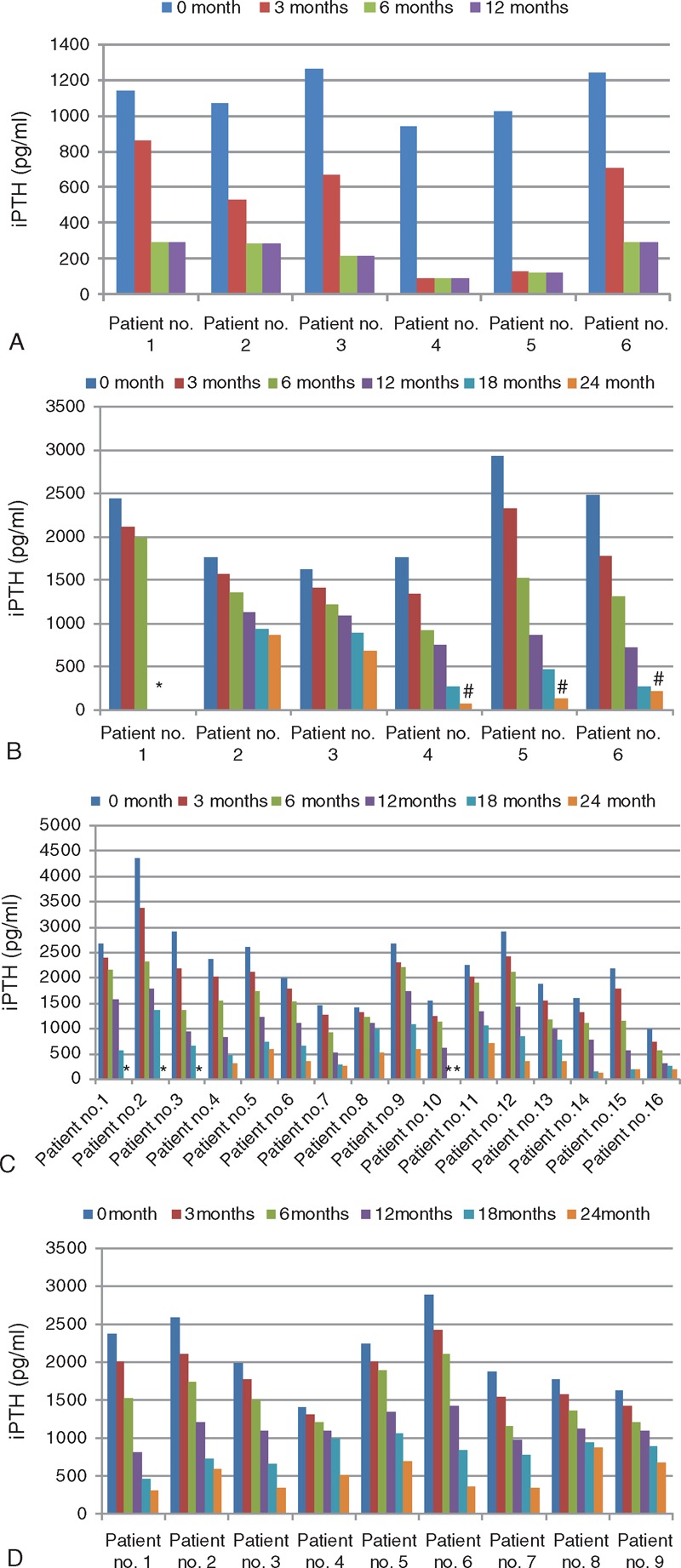
(A) Different iPTH levels in patients with CKD-4 at different cinacalcet doses. (B) Different iPTH levels in patients with CKD-5 on hemodialysis at different cinacalcet doses. (C) Different iPTH levels in patients with CKD-5 on peritoneal dialysis at different cinacalcet doses. (D) Different iPTH levels in partially responding patients with CKD-5 at different cinacalcet doses. CKD = chronic kidney disease, iPTH =  intact parathyroid hormone.

Table 2 demonstrates patients’ s.iPTH level 1 month pre-treatment and at the end-point of the study. All patients showed a minimum reduction in s.iPTH level of 60% (range 60%–97%) over a period of continuous treatment for 3 to 24 months until the end-point is achieved. Six patients died of CKD: 2 from the HD group and 4 from APD group. Nine patients still did not achieve the end-point at 24 months and are still on treatment (Table 2 and Figure 1).

**Table 2:**
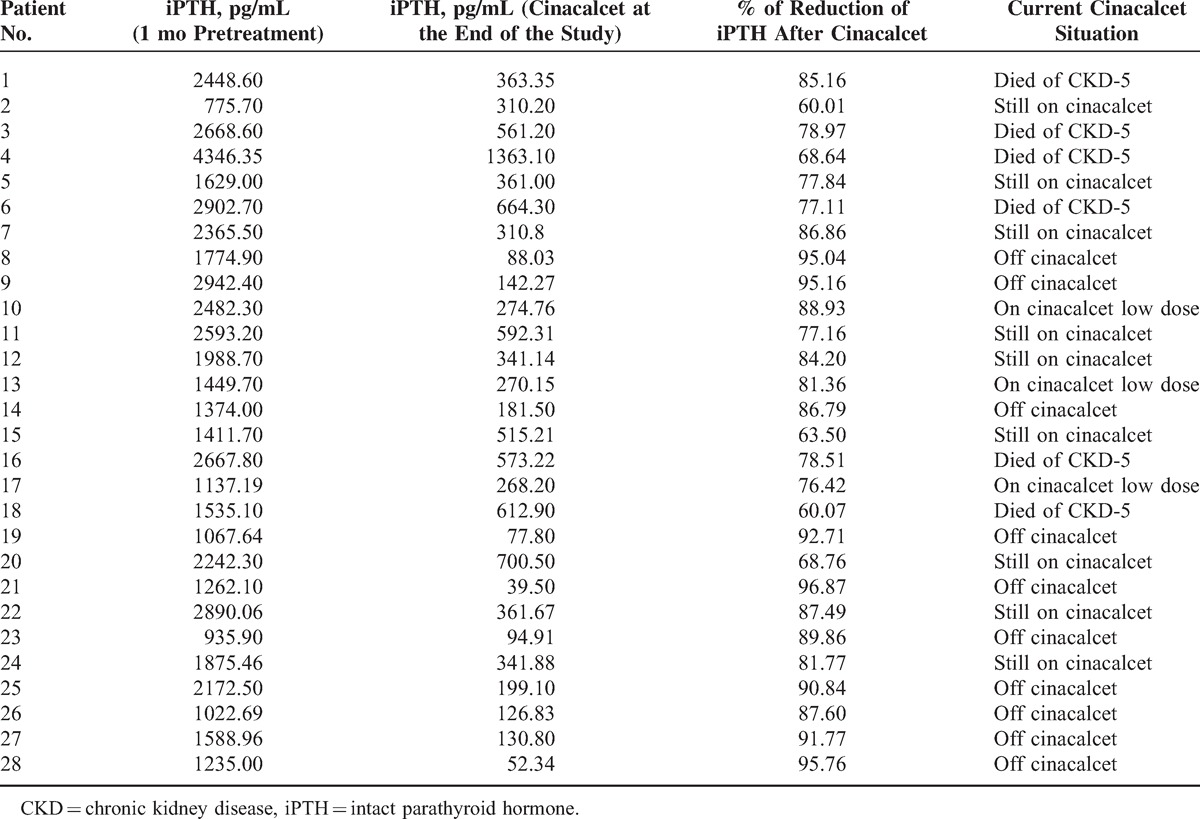
Serum iPTH Values Pretreatment and Posttreatment With Cinacalcet

**Figure 2:**
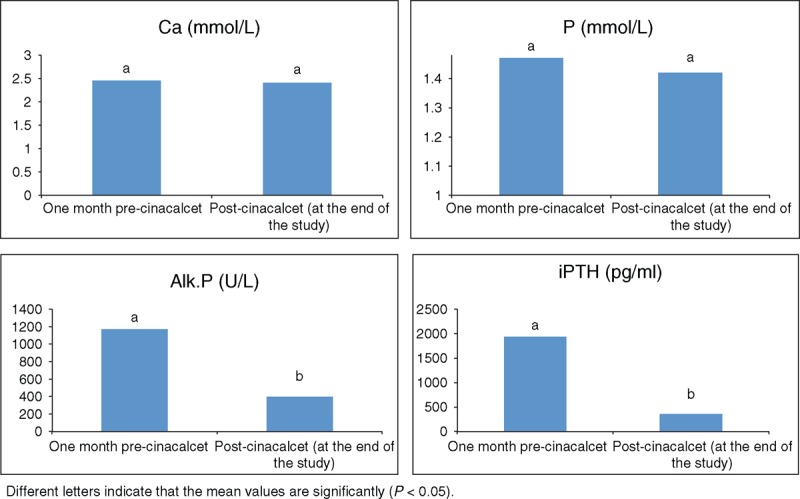
Effect of cinaclacet on bone biochemistry.

Highly significant reduction in s.iPTH and s.Alk.P levels was demonstrated with cinacalcet treatment (*P* < 0.001). No significant difference in s.Ca and s.P or urinary excretion of Ca, P, and proteins was detected over the duration of treatment with no symptomatic hypocalcemia, hypophosphatemia, or other adverse side effects including soft tissue, vascular, or renal calcification with negative renal U/S and echocardiography evidence of calcification all through the study period (Table 3 and Figure 2).

**Table 3:**
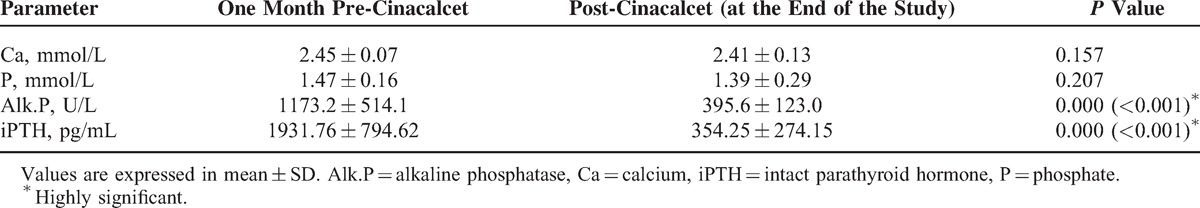
Serum Ca, P, and iPTH Levels Pre-Cinacalcet and Post-Cinacalcet Use

These data support not recommending frequent regular examinations like renal U/S and echocardiography as screening tests during cinacalcet treatment. They can be done annually as supported by the negative results all through the 24-month duration of our study.

An interesting finding in the present study is that growth parameters did not deteriorate by cinacalcet management. Another interesting point is that CKD-4 patient sustained a static course of their renal disease without progression toward end-stage renal disease. These findings need larger studies to validate their significance.

## DISCUSSION

Studies on cinacalcet in pediatric CKD-4/5 are scarce. Some case reports^22,23^ and only 4 pediatric prospective studies^2,24–26^ were conducted on a small number of patients (largest was on 9 patients), mostly adolescents, the youngest was 11 months, treated for periods ranging from 1 month to 3 years. They showed a positive effect of cinacalcet on short-term iPTH suppression,^2,22–25^ with one small study reporting rebound hyperparathyroidism.^26^ Two studies reported hypocalcemia.^24,25^ The summary of the published Pediatric studies and case reports is shown in Table 4.

**Table 4:**
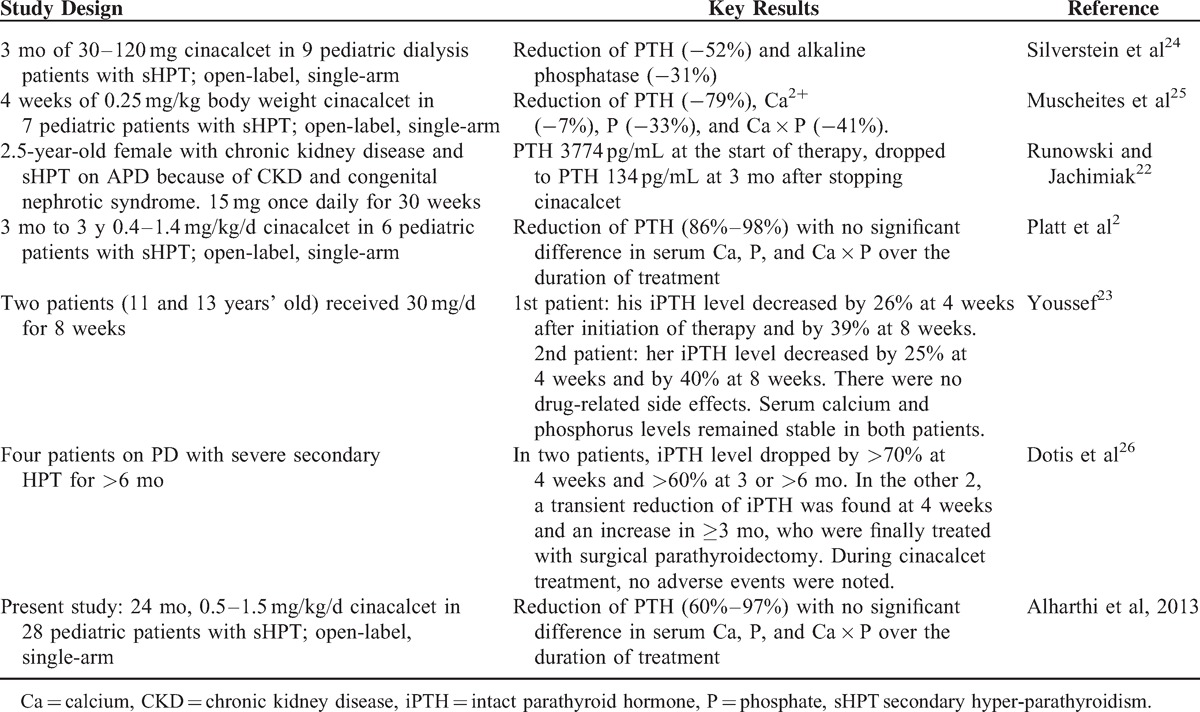
Summary of Clinical Studies on Cinacalcet in Pediatric CKD

The results of the present study are in agreement with other studies^2,24,25^ in demonstrating a marked decrease in s.iPTH levels with cinacalcet treatment. Our data demonstrated that this effect can be sustained. This study adds to the existing knowledge that cinacalcet can cause decrease in s.Alk.P plus its high efficacy in CKD-4 and potential safety and effectiveness in young infants.

Although there are clear benefits of cinacalcet use as supported by adult studies, it remains a concern about the potential for a negative impact on growth in children. There are presently no experimental studies to support this concern. The CaSRs are expressed on chondrocytes at the growth plates of epiphyses and have a role in the proliferation and differentiation of these cells in vitro.^27,28^ A recent study in uremic rats has shown no negative impact on growth-plate chondrocyte function with cinacalcet use.^28^ The effects of modulating these receptor during active periods of skeletal growth throughout childhood are unknown and require careful evaluation. The time period over which we used cinacalcet was 24 months and still ongoing in 9 patients, during which no major adverse impact on linear growth was observed. It is therefore felt reassuring that cinacalcet may not affect linear growth; however, larger studies for a longer duration are needed to prove or disprove this issue.

Previous adult studies have shown a simultaneous reduction in s.iPTH, s.Ca, and s.P on response to cinacalcet therapy.^17,29^ Its effect on mineral homeostasis is less clear in pediatric CKD population, perhaps as a result of active skeletal growth and the consequent influx and efflux of these minerals from bones.^10,30^ Pediatric studies were nonconclusive in that respect, with one study (over a 4-week period) showing a significant decrease in the median level of s.P and Ca × P product.^24^ A second more lengthy study (over a 3-month period) demonstrated no effect on s.Ca, s.P, or Ca × P product despite an overall significant reduction in s.iPTH levels.^25^

Platt et al^2^ agreed with us in that cinacalcet can have clinically significant effects on s.Ca and s.P in the short term, necessitating alterations in medication regimens.

Highly significant reduction in s.Alk.P levels post-cinacalcet was detected. Silverstein et al^24^ also demonstrated decline of s.Alk.P levels in their cohort.

The addition of cinacalcet to CKD-4/5 conventional management was shown to be helpful in controlling apparently refractory levels of s.iPTH without side effects, which in all cases may have resulted in avoidance of parathyroidectomy. This agrees with adult studies that recommend a combination of low-dose cinacalcet in combination with conventional management over conventional management alone for control of CKD secondary hyperparathyroidism.

An important finding is that initiating cinacalcet treatment at lower levels of s.iPTH between 300 and 500 pg/mL in addition to traditional therapy enabled 80% of our patients to achieve iPTH treatment goal (s.iPTH ≤300 pg/mL) within 6 months, whereas some of those in whom cinacalcet was started on much higher levels of s.iPTH >800 pg/mL achieved the same goal after 24 months and some of them did not achieve at all.

## CONCLUSION

In pediatric CKD-4/5, cinacalcet management appears to be effective in suppressing s.iPTH and achieving control over bone biochemistry facilitating the resolution of hyperparathyroidism resistant to conventional treatment in most of patients.

Parathyroidectomy, a complicated and technically challenging procedure in children, can be avoided.

Among pre-dialysis pediatric patients with secondary hyperparathyroidism that is refractory to therapy with Vita D analogues, Ca supplements, and P binders, cinacalcet was safe and effective without any significant complications as long as close monitoring of bone biochemistry and dosage adjustment are implemented (our maximum dose among this group was 0.5 mg/kg/d). Changeable doses of active Vit D are mandatory throughout cinacalcet treatment.

Further studies are needed to verify the effects of cinacalcet on linear growth in children. We recommend cinacalcet use on a wide scale in pediatric CKD-4/5 even at young age. Experimental evidence suggests that calcimimetic agents may impede the development of parathyroid gland hyperplasia, an integral component of secondary hyperparathyroidism, due to chronic renal failure. Future studies will probably show whether an earlier start of treatment in patients with CKD stage 2 and 3 allows the prevention of secondary hyperparathyroidism.
